# The cGAS–STING Pathway in Dementia: An Emerging Mechanism of Neuroinflammation

**DOI:** 10.3390/brainsci15111241

**Published:** 2025-11-19

**Authors:** Young Min, Yoon-Seob Lee, Juwon Lee, Da-Young Keum, Joo-Young Gwag, Sung-Min Jeon, Heejin Jo, Sung-Ung Kang

**Affiliations:** 1 Department of Molecular and Cell Biology, University of California, Berkeley, CA 94720, USA; youngm020907@gmail.com; 2 Department of Dentistry, College of Dentistry, Yonsei University, Seoul 03722, Republic of Korea; 3 The Yonsei Dental Clinic, Suwon 16385, Republic of Korea; 4 Neuroregeneration and Stem Cell Programs, Institute for Cell Engineering, Johns Hopkins University School of Medicine, Baltimore, MD 21205, USA; japple3.141592@gmail.com (J.L.); dykeum002@gmail.com (D.-Y.K.); samuelchun08@gmail.com (S.-M.J.); 5 Department of Biomolecular Engineering and Bioinformatics, University of California, Santa Cruz, CA 95064, USA; luminousardor1010@gmail.com; 6 Department of Korean Medicine, CHA University School of Medicine, Seoul 06062, Republic of Korea; 7 Department of Neurology, Johns Hopkins University School of Medicine, Baltimore, MD 21205, USA

**Keywords:** neurodegeneration, dementia, Alzheimer’s disease, immune response, disease associated microglia, cGAS-STING pathway

## Abstract

Dementia is a growing global health concern in aging societies, leading to a progressive decline in cognitive function that severely impairs daily life. Despite the growing burden, effective preventive and therapeutic strategies remain elusive, emphasizing the urgent need for novel interventions. Recent advances underscore the pivotal role of neuroinflammation in dementia pathogenesis, particularly in Alzheimer’s disease (AD). Chronic activation of central nervous system immune cells, particularly microglia, exacerbates neurodegeneration and establishes a self-perpetuating cycle of inflammation and cognitive decline. This review focuses on emerging research exploring the cGAS-STING pathway’s role in dementia, examining its potential as a diagnostic and therapeutic target. The cGAS-STING pathway, integral to innate immune responses, may contribute to the chronic neuroinflammation seen in neurodegenerative diseases. By targeting this pathway, new strategies could mitigate the inflammatory processes that drive neuronal loss, offering a promising avenue for therapeutic development in dementia.

## 1. Introduction

Dementia is a clinical syndrome defined by gradual impairment of memory, executive function, language, and visuospatial skills, leading to loss of independence while typically sparing basic alertness or consciousness. At its core, dementia is driven by the loss of neurons and synaptic connections within the brain, which disrupts neural circuits necessary for cognitive integrity [1]. The incidence of dementia has risen significantly due to global aging trends, with an increase in cases from 20.2 million in 1990 to 43.8 million in 2016 [2]. Projections estimate that by 2050, the number of people living with dementia will escalate to over 152.8 million globally, reflecting both increased life expectancy and the persistent lack of effective preventive measures [3,4].

Recent advances in our understanding of dementia pathogenesis have highlighted the significant contribution of neuroinflammation to disease progression, particularly in Alzheimer’s disease (AD) and other neurodegenerative conditions [5]. Neuroinflammation is characterized by the activation of immune cells within the central nervous system (CNS), notably microglia and astrocytes, which can release pro-inflammatory cytokines, chemokines, and reactive oxygen species (ROS). While acute inflammation is necessary for CNS defense and tissue repair, engaging resident immune cells to clear pathogens such as viruses and bacteria and to restore homeostasis [6], chronic neuroinflammation is implicated in the exacerbation of neuronal damage, ultimately leading to cognitive decline [7]. Microglia, the resident immune cells of the CNS coupled with the activation of inflammatory signaling pathways, exacerbates the progression of AD and other dementias. In response to pathogenic stimuli such as amyloid-β (Aβ) plaques or tau aggregates, microglia undergo a phenotypic transformation from a homeostatic to a pro-inflammatory state, releasing a cascade of inflammatory mediators [8].

Additionally, the cGAS-STING pathway is increasingly seen as a major player in mediating these inflammatory responses, linking cytosolic DNA sensing to neuroinflammatory cascades [9,10,11,12,13]. Although no cure exists for AD or other forms of dementia, targeting neuroinflammatory signaling such as modulating microglial activation, inhibiting pro-inflammatory cytokines, or suppressing the cGAS-STING pathway has emerged as a promising therapeutic strategy for their potential to slow disease progression and preserve cognitive function [9,14]. Accordingly, this review summarizes current understanding of neuroinflammation and highlights emerging evidence for the cGAS-STING pathway as a diagnostic and therapeutic target in dementia.

## 2. Neuroinflammation in Dementia: A Shared Mechanism

Dementia is an umbrella term encompassing a range of cognitive impairments that significantly affect memory, thinking, and behavior. AD, the most common form of dementia, accounts for 60–80% of all dementia cases [15]. While all individuals with Alzheimer’s have dementia, not all individuals with dementia have AD, as dementia can arise from various etiologies, including vascular causes, Lewy body formations, or frontotemporal degeneration [16], distinguished by the type of protein aggregation and its localization in the brain. AD, Vascular Dementia (VaD), Lewy Body Dementia (LBD), and Frontotemporal Dementia (FTD)—are associated with distinct pathophysiological mechanisms, yet all are linked to chronic neuroinflammatory processes, which exacerbate neuronal damage and cognitive deficit [17,18].

### 2.1. Disease-Specific Mechanisms and Neuroinflammatory Pathways in Dementia

In AD, the most common form of dementia, the classical hallmark features include the accumulation of amyloid-β (Aβ) plaques and tau tangles. These aggregates primarily affect the medial temporal lobe and neocortical regions, leading to synaptic dysfunction, neuronal death, and eventual brain atrophy [16,19]. Aβ and tau accumulation is believed to trigger a neuroinflammatory cascade of events, including the activation of microglia, the resident immune cells of the central nervous system, and the release of pro-inflammatory cytokines such as Interleukin-1α(IL-1α), Interleukin-1β (IL-1β), Tumor Necrosis Factor-alpha (TNF-α), Interleukin-6(IL-6), Interleukin-12(IL-12) and interleukin-23(IL-23) and chemokines such as Chemokine CC motif Ligand 2(CCL2), Chemokine CXC motif Ligand 8(CXCL8 or IL-8), CCL3, CCL4, and Macrophage Inflammatory Peptide-1 (MIP-1α), which further exacerbate neuronal damage [5,20,21]. VaD, the second most common type of dementia, results primarily from cerebrovascular injury, such as cortical infarctions and white matter lesions. The subtypes of VaD include post-stroke dementia (PSD), multi-infarct dementia (MID), and subcortical ischemic vascular dementia (SIVD) [22]. Recent evidence suggests that not only ischemia-induced neuronal damage but also neuroinflammation plays a pivotal role in VaD progression. In particular, pro-inflammatory cytokines such as IL-6 and TNF- α, as well as activated microglia, are known to disrupt the blood–brain barrier (BBB) exacerbate cerebral ischemia and contribute to the progressive loss of white matter integrity [23,24], triggering inflammatory responses and neuronal injury that contribute to cognitive decline [25]. Chronic neuroinflammation in VaD has also been associated with systemic risk factors such as hypertension and diabetes, further implicating the immune system in the progress of vascular brain damage [26]. LBD, on the other hand, is characterized by the aggregation of α-synuclein into Lewy bodies, predominantly affecting the brainstem, cortex, and limbic regions. The pathogenesis of LBD is complex, driving by an interplay of genetic risk factors such as APOE e4 allele, triggering receptor expressed on myeloid cells 2 (TREM2) and glucocerebrosidase (GBA) and acquired factors like aging, systemic inflammation and altered microbiota. Activated microglia surrounding Lewy bodies release pro-inflammatory cytokines such as IL-1β and TNF-α, which may contribute to neuronal death and synaptic dysfunction [27,28]. Elevated neuroinflammatory markers correlate the severity of cognitive and motor impairments in LBD patients [28,29]. FTD involves progressive degeneration of the frontal and temporal lobes, with marked neuronal atrophy linked to proteinopathies such as tau or TDP-43 aggregates which also stimulate neuroinflammatory pathway [30]. Neuroimaging studies have revealed that increased microglial activation in patients with FTD and correlates with disease progression and severity of symptoms [30,31,32].

Despite differing etiologies, the convergence of microglial activation and neuroinflammation forms a unifying theme in dementia pathogenesis. In each case, microglial activation contributes to the release of pro-inflammatory cytokines, oxidative stress and chronic neurodegeneration [33]. Chronic systemic inflammation, stemming from infections, lifestyle factors, or aging itself, may influence microglial priming and exacerbate neurodegenerative processes [7,34]. Biomarkers and neuroimaging studies reinforce this shared mechanism. In AD, elevated levels of pro-inflammatory cytokines, including interleukin-6 (IL-6) and tumor necrosis factor-alpha (TNF-α), have been detected in both the CSF and plasma [35]. Additionally, elevated soluble TREM2 (sTREM2), explored as a marker of microglial activation [36], correlate with neuroinflammatory activity and may predict the progression of AD [36,37]. In FTD, increased levels of progranulin and inflammatory cytokines such as IL-8 are being investigated as potential biomarkers of microglial dysfunction [30]. Similarly to traditional biomarkers, inflammatory biomarkers in the CSF remain difficult to utilize clinically due to the invasive nature of lumbar punctures, efforts are being made to identify reliable blood-based biomarkers. Plasma levels of sTREM2 and cytokines, as well as microRNA profiles, have shown potential in reflecting neuroinflammatory processes in both AD and FTD.

Neuroimaging studies have evolved beyond structural assessments and now allow the visualization of neuroinflammation in vivo. Positron Emission Tomography (PET) using ligands that target the translocator protein (TSPO), which is upregulated in activated microglia, has emerged as a powerful tool for mapping neuroinflammation in various forms of dementia [38]. In AD, TSPO-PET imaging has shown increased microglial activation in regions corresponding to amyloid-β deposition, particularly in the hippocampus and frontal cortex [38,39]. Similarly, in FTD, TSPO binding correlates with microglial activation in regions undergoing neurodegeneration, such as the frontal and temporal lobes [32]. This technique also holds promise in VaD, where chronic neuroinflammation in response to cerebrovascular damage has been observed using TSPO-PET. Furthermore, advanced MRI techniques such as diffusion tensor imaging (DTI) and arterial spin labeling (ASL) are being used to assess white matter integrity and cerebral blood flow, respectively, in the context of neuroinflammatory damage. In LBD, neuroinflammation visualized through these techniques may help differentiate the disease from other neurodegenerative disorders [27].

### 2.2. Genetic Drivers of Neuroinflammation in Dementia

Recent advances in genetic research have yielded significant insights into the genetic basis of dementia,. highlighting the role of genetic variants in inflammatory pathways. Genome-wide association studies (GWAS) continue to identify novel loci associated with dementia subtypes, highlighting the complex interplay between genetic predispositions, immune function and neuroinflammatory processes in dementia [40,41].

One of the most well-known genetic risk factors for AD is the apolipoprotein E ε4 allele (APOE-ε4), which not only influences amyloid-β deposition but also alters microglial reactivity. APOE-ε4 carriers exhibit increased expression of pro-inflammatory cytokines in the presence of amyloid plaques, exacerbating neurodegeneration [42,43]. Variants in the triggering receptor expressed on myeloid cells 2 (TREM2) gene, which modulates microglial activation, have been identified in populations with high dementia risk and associated with an increased risk for late-onset AD. TREM2 mutations impair the microglial response to amyloid-β, resulting in inefficient clearance of toxic aggregates and heightened neuroinflammation [44]. This impaired microglial response to inflammatory stimuli and phagocytosis exacerbates neurodegeneration in FTD as well [44]. The sialic acid-binding immunoglobulin-like lectins (CD33) and the membrane-spanning 4-domain subfamily A (MS4A) gene families suggest additional connections to microglial signaling. CD33 variants particularly regulate the immune inhibitory receptor functions of microglia, linking them to increased neuroinflammation [45,46]. ATP-binding cassette transporter subfamily A member 7 (ABCA7) is also known to alter the response of inflammation, leading to amyloid accumulation [47]. Variants in the MS4A gene cluster, which regulate immune signaling, are associated with AD risk [40,48]. Furthermore, in FTD, mutations in the microtubule-associated protein tau (MAPT)gene have been shown to drive neuroinflammation by promoting tau pathology and inducing a reactive microglial state [49]. Mutations in progranulin gene (GRN), a known risk factor for familiar FTD, have been shown to modulate microglial activation and the production of pro-inflammatory cytokines, amplifying neuroinflammatory responses [30,50]. Similarly, C9orf72 gene disrupt autophagy and immune regulation, resulting in increased neuroinflammation and microglial activation in FTD [51]. In LBD, genes involved in α-synuclein processing, such as SNCA, also have indirect ties to neuroinflammation through their effects on microglial activation [41] (Table 1).

## 3. cGAS-STING Pathway and Neuroinflammation in Dementia: A Potential Therapeutic Target

### 3.1. Molecular Mechanisms of cGAS-STING Pathway

**Figure 1:**
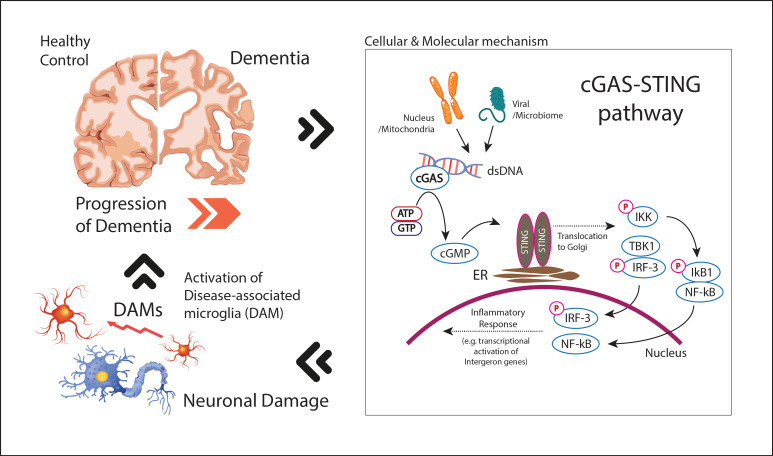
The cGAS-STING pathway in dementia progression and microglial activation Cytosolic DNA activates cGAS to synthesize cGAMP, which binds STING and triggers TBK1-IRF3 and NF-κB signaling. Both pathways induce the transcriptional activation of pro-inflammatory genes and interferons, amplifying the inflammatory response and contributing to neuronal damage in dementia by the activation of disease-associated microglia (DAMs). This feedback loop between DAMs and neuroinflammation potentially exacerbates the progression of dementia. cyclic GMP–AMP (cGAMP), TANK-binding kinase 1 (TBK1), Interferon regulatory factor 3 (IRF3), Nuclear factor kappa-light-chain-enhancer of activated B cells (NF-κB), Inhibitor of κB alpha (IKB1, NFKBIA, IκBα), IκB kinase complex (IKK).

The cGAS-STING pathway represents a crucial component of the body’s innate immune system, functioning primarily as a sensor for cytosolic DNA. Cyclic GMP-AMP synthase (cGAS) detects aberrant DNA within the cytoplasm—whether of exogenous origin, such as viral or bacterial DNA, or endogenous, such as mitochondrial or nuclear DNA released during cellular stress or damage. Upon binding to this DNA, cGAS catalyzes the synthesis of cyclic GMP-AMP (cGAMP), a second messenger that subsequently binds to and activates STING (Stimulator of Interferon Genes) [64]. This activation triggers downstream signaling through the TANK-binding kinase 1 (TBK1), leading to the phosphorylation of the transcription factor interferon regulatory factor 3 (IRF3) and the subsequent induction of Type I interferon (IFN) and other pro-inflammatory cytokines [33] (Table 2, Figure 1).

### 3.2. cGAS-STING Activation in Dementia

In the context of neurodegenerative diseases, such as AD and other types of dementia, the activation of the cGAS-STING pathway has gained increasing attention as a key mediator of neuroinflammation [9,14]. Mitochondrial dysfunction, which is well established in neurodegenerative conditions, can lead to the release of mitochondrial DNA (mtDNA) or double-stranded DNA (dsDNA) into the cytoplasm, where it serves as a potent activator of cGAS [33]. This activation may exacerbate chronic neuroinflammation, a hallmark of neurodegenerative diseases. Additionally, nuclear DNA instability, which may occur during cellular senescence or in the presence of oxidative stress, can also trigger the cGAS-STING pathway, further promoting neuroinflammatory responses (Figure 1).

Emerging evidence further suggests that cGAS-STING activation is involved in the progression of several neurodegenerative diseases. Both cGAS and STING protein levels are substantially elevated in the brains of 5xFAD mice, the most commonly used transgenic AD model. Genetic deletion of cGAS or STING in 5xFAD shows reduced Aβ plaque, altered microglial activation with reduced pro-inflammatory gene expression and protected cognitive function [65,66]. In Parkinson’s disease (PD), mouse models of α-synucleinopathies, which mimic the neuropathology of PD, show specific activation of cGAS-STING in the nigrostriatal regions, accompanied by elevated cytokine levels and enhanced neuroinflammation [67]. In amyotrophic lateral sclerosis (ALS), TDP-43 protein aggregates have been found to disrupt mitochondrial integrity, leading to mtDNA release and subsequent cGAS-STING activation [68]. A similar phenomenon has been observed in Huntington’s disease (HD), where cGAS upregulation has been identified in both murine models and human tissues [14,69].

Beyond these specific diseases, the cGAS-STING pathway also appears to play a significant role in broader neuroinflammatory processes. Elevated type I interferon levels, a downstream consequence of STING activation, have been documented in models of prion disease, promoting microglial activation and perpetuating neuroinflammatory cascades [33]. Post-mortem analyses of human CNS tissues from patients with AD, PD, ALS, and multiple sclerosis (MS) reveal increased STING protein levels in neurons and brain endothelial cells [70,71,72]. In vitro studies further demonstrate that mitochondrial stress induced by factors like palmitic acid leads to cytosolic DNA leakage and robust activation of the cGAS-STING axis, suggesting that metabolic dysfunction may be a common trigger of neuroinflammation [73,74].

## 4. Translational Insights: Therapeutic Targeting of cGAS-STING in Neurodegeneration

Current therapeutic approaches for dementia remain largely symptomatic and do not adequately address the underlying neuroinflammatory pathophysiology. The predominant treatments, including cholinesterase inhibitors and NMDA receptor antagonists, provide modest symptomatic benefits but do little to alter the disease progression. This limitation stems from the complex etiology of dementia, wherein neuroinflammation plays a pivotal role in driving neuronal loss and disease progression [75].

Among the inflammatory pathways implicated, the cGAS-STING pathway has emerged as a critical mediator linking cytosolic DNA sensing to innate immune activation. Its overactivation has been associated with chronic neuro-inflammation and neurodegeneration, making it a promising therapeutic target. Current strategies to inhibit this pathway focus on both cGAS, the cytosolic DNA sensor, and STING, the adaptor proteins.

### Therapeutic Modulation of cGAS-STING Pathway: Comparison Between Targeting cGAS vs. STING

cGAS inhibitors aim to block the synthesis of cGAMP, thereby reducing STING activation and downstream interferon production [76]. These inhibitors typically target the active site of cGAS or disrupt its interaction with dsDNA. Although several small-molecule inhibitors have shown promise in preclinical models, none have advanced to clinical trials [77].

Most STING inhibitors, by contrast, aim to block the ligand-binding domain or interfere with post-translational modifications that enhance STING activity [76]. Their potential in neurodegenerative diseases remains largely unexplored while it has been focused in the context of cancer immunotherapy [76]. Despite the lack of clinically approved inhibitors, research in this area continues to evolve, offering hope for future therapies targeting neuroinflammation in dementia and related disorders.

Targeting cGAS versus STING offers distinct points of intervention within the same signaling cascade, yet the optimal target remains uncertain. Whether inhibiting the upstream sensor, cGAS, or the downstream adaptor, STING, yields superior efficacy or safety has yet to be determined as well, as both act sequentially in DNA-initiated signaling. However, targeting cGAS or STING is generally preferred rather than targeting downstream components such as TBK1 or IFNAR as it allows other pattern recognition receptor systems remain functional.

Although no clinically approved inhibitors currently exist, continued research into selective and tunable modulators of the cGAS–STING pathway offers a promising avenue for developing disease-modifying therapies in dementia and related neurodegenerative disorders. The compounds which have demonstrated efficacy in preclinical neurodegenerative models by targeting the cGAS-STING axis are shown in Table 3.

## 5. Conclusions

The recognition of cGAS-STING pathway as an upstream axis related to chronic neuroinflammation indicates a deeper understanding in neurodegenerative disease. In summary, the cGAS-STING pathway plays a crucial role in mediating neuroinflammation across various dementia including AD, VaD, PD and FTD. Activation of this pathway, often triggered by mitochondrial or nuclear DNA leakage, initiates a pro-inflammatory cascade, involving IFN-I signaling predominantly in microglia but also in vulnerable neurons and brain endothelial cells. Consequently, it accelerates neuronal damage and disease progression. The connection between this pathway and major risk factors for dementia such as *APOE*, *TREM2*, *c9orf72* highlights cGAS-STING pathway as a common convergence point in neuro-immune axis, affecting disease onset and progression.

While much remains to be understood about the precise mechanisms by which cGAS-STING contributes to neurodegeneration, the development of inhibitors targeting this pathway has already demonstrated preclinical success in alleviating neuroinflammation and pathology in dementia animal models. For clinical translation, the exploration of these inhibitors should consider blood–brain barrier penetration because species variability, for example, molecular differences between murine STING and human STING, could yield different permeability and efficacy. Also, potential systemic immune suppression should also be considered as cGAS-STING pathway is essential for host defense. Validating specific biomarkers of cGAS-STING activation such as measuring cGAMP or STING expression and developing new neuroimaging techniques such as new PET tracers visualizing the activation would be essential for identifying and monitoring the disease state and should be considered in the future research.

## Figures and Tables

**Table 1:** Genetic mutations associated with dementia that are also linked to microglial activation.

Gene Symbols	Full Name	Chromosomal Location	Role in Microglia Activation	Associated Dementia	References
***Genes governing microglial activation and phagocytosis***					
*TREM2*	Triggering Receptor Expressed on Myeloid cells 2	6p21.1	Impair microglial activation, reduce phagocytosis of Aβ and damaged cells.	AD, FTD	[52,53]
*APOE ε4*	Apolipoprotein E (Allele ε4)	19q13.32	impairs microglial Aβ clearance; detrimental TREM2 signaling pathway driving microglia from a resting state to a protective	AD	[54]
*CD33*	Cluster of Differentiation 33	19q13.41	Inhibitory signal that suppresses microglial phagocytosis of Aβ	AD	[45,46]
*PLCG2*	Phospholipase C Gamma 2	16q23.3	downstream effector of TREM2, promote microglial activation and phagocytosis, improving immune response in AD.	AD	[55]
*MAPT*	Microtubule-associated Protein Tau	17q21.32	Promotes tau pathology, induce reactive microglial state	FTD	[49]
***Genes involved in protein and debris clearance within the microglia***					
*GRN*	Granulin (progranulin)	17q21.31	Lysosomal dysfunction, excessive microglial activation, impaired microglia debris clearance	FTD	[50]
*SORL1*	Sortilin Related Receptor 1	11q24.2	lysosome dysfunction of microglia, malfunction in processing and trafficking of amyloid precursor protein and Aβ	AD	[56]
*TMEM106B*	Transmembrane Protein 106B	7p21.3	lysosomal dysfunction in microglia, excessive microglial activation, impaired waste clearance.	FTD	[57]
*ABCA7*	ATP Binding Cassette Subfamily A Member 7	19p13.3	abnormal cholesterol efflux and lipid metabolism, impaired microglial phagocytosis and inflammatory response	AD	[47]
*PS1 (PSEN1)*	Presenilin 1	14q24.2	Cause massive Aβ burden leading secondary microglial toxicity, impaired lysosomal calcium signaling and autophagy leading abnormal brain homeostasis	AD	[58]
*SNCA*	Synuclein Alpha	4q22.1	Lipid peroxidation, loss of antioxidant defense, microglial phagocytic exhaustion	LBD	[41]
***Genes affecting complement and inflammatory signaling that microglia use to label and clear damaged cells***					
*CR1*	Complement Receptor type 1	1q32.2	Variants in CR1 impair complement regulation and enhance microglial activation via the complement cascade.	AD	[59]
*MS4A*	Membrane Spanning 4 domain, subfamily A	11q12.2	Altered membrane protein interaction, abnormal microglial inflammatory signaling	AD	[40,48]
*BIN1*	Bridging Integrator 1	2q14.3	The second most significant AD risk gene, highly expressed in microglia, Abnormal microglial phagocytosis and endocytosis of Aβ	AD	[60]
*LILRB2*	Leukocyte Immunoglobulin-Like Receptor B2	19q13.42	co-expressed with TREM2 in microglia, inhibitory receptor, suppresses microglial phagocytosis of Aβ	AD	[61]
***Genes associated with a non-cell-autonomous role contributing to neurotoxicity***					
*C9orf72*	Chromosome 9 open reading frame 72	9p21.2	dysregulated microglial activation, lysosomal storage defects, toxic inflammatory activation	FTD, ALS	[51]
*MEF2C*	Myocyte Enhancer Factor 2C	5q14.3	A transcriptional factor, modulating inflammatory environment in microglia	AD	[62]
*SOD1*	Superoxide Dismutase 1	21q22.11	increases oxidative stress, enhanced toxic microglial activation	ALS	[63]

**Table 2:** Key Molecular Components of cGAS-STING Pathway.

Component	Cellular Location/Target	Function in Activation	Primary Downstream Effect
cGAS	Cytosol /Nucleus	Recognizes cytosolic dsDNA (DAMPs/PAMPs); dimerizes/catalyzes cGAMP synthesis	cGAMP production
cGAMP	Cytosol	Second messenger; binds and activates STING protein	STING conformational change and trafficking (from ER to Golgi)
STING	Endoplasmic Reticulum	Activated by cGAMP; recruits and activates TBK1	Recruitment and phosphorylation of TBK1
TBK1	Cytosol /Golgi	Kinase; Phosphorylates STING, IRF3, and IKK	Activation of IRF3/NF-κB pathways
IRF3	Cytosol /Nucleus	Transcription Factor; drives transcription of inflammatory genes	Transcription of Type I IFNs

**Table 3:** Pharmacological Inhibitors of the cGAS-STING pathway in dementia.

Compound Name/Class	Target	Mechanism of Action (MOA)	Key Preclinical Use/Disease Model	References
RU.521, RU.365 (Tricyclic benzofluoropyrimidine compounds)	cGAS	Inhibit cGAS catalytic activity by competitive binding to DNA-binding domain; blocks dsDNA-cGAS interaction and cGAMP synthesis	subarachnoid hemorrhage(SAH)/macrophages from autoimmune mice Trex1^−/−^ mice, human iPSC-derived Huntington’s disease (HD) neurons, ed in iPSC-derived ALS neurons/cGAS inhibition, Neuroprotection, reduced cognitive dysfunction, microglial inflammation	[69,76,78]
A151	cGAS	Inhibitor that competes with dsDNA for cGAS binding; Synthetic oligonucleotide antagonist	Acute brain trauma/mouse model of ischemic stroke; reduced neuronal loss, immune cell infiltration and infarct volume	[14,76]
TDI-6570	cGAS	Brain-permeable cGAS inhibitor	AD/P301S tauopathy mouse model/protect impaired cognitive and synaptic function	[9]
H-151	STING	Covalent binding to Cys91; blocks palmitoylation/clustering (irreversible)	AD, ALS, FTD/5xFAD mice, TDP-43 mutant mice, iPSC-derived ALS neurons/reduced phospho-IRF3, improved cognitive function in aged mice	[71,72,76]
,SN-011	STING	Potent small molecule inhibitor	Aicardi–Goutières syndrome (AGS)/Trex1-deficient model/Mitigating neurological impairment)	[78]

## Data Availability

No new data were created or analyzed in this study.
